# Sex-specific role of microglia in Δ^9^-tetrahydrocannabinol-induced disruption of fear memory reconsolidation

**DOI:** 10.1038/s41386-026-02434-x

**Published:** 2026-05-09

**Authors:** Ana Maria Raymundi, Nathalie Carla Cardoso, Gabriel Costa Lourenço, Ruliam Queiroz, Maressa D. Dolzan, Luciano Vitali, Erika Meyer, Sabrina Francesca Lisboa, Francisco Silveira Guimarães, Graziano Pinna, Leandro José Bertoglio, Cristina Aparecida Jark Stern

**Affiliations:** 1 Department of Pharmacology, Federal University of Paraná, Curitiba, PR Brazil; 2 Federal Institute of Santa Catarina, Itajaí, SC Brazil; 3 Department of Chemistry, Federal University of Santa Catarina, Florianópolis, SC Brazil; 4 Department of BioMolecular Sciences, School of Pharmaceutical Sciences of Ribeirão Preto, University of São Paulo, Ribeirão Preto, SP Brazil; 5 Department of Pharmacology, Ribeirão Preto Medical School, University of São Paulo, Ribeirão Preto, SP Brazil; 6 The Psychiatric Institute, Department of Psychiatry, College of Medicine, University of Illinois Chicago, Chicago, IL USA; 7 Department of Pharmacology, Federal University of Santa Catarina, Florianópolis, SC Brazil

**Keywords:** Fear conditioning, Behavioural methods, Neurophysiology

## Abstract

In PTSD, microglia are more engaged in fear-related circuits, where they participate in fear memory processing responses. The reconsolidation-impairing effect of Δ⁹-tetrahydrocannabinol (THC), the primary psychoactive constituent of Cannabis, is typically linked to cannabinoid type-1 receptor (CB1) signaling. THC also engages peroxisome proliferator-activated receptor gamma (PPARγ), and both CB1 and PPARγ modulate neuroimmune processes, including microglial activity. This dual mechanism suggests that THC’s impact on fear memory reconsolidation may extend beyond classical cannabinoid signaling. We hypothesize that THC disrupts contextual fear memory reconsolidation via microglial recruitment in the dorsal hippocampus (DH) through a sex-specific engagement of CB1 and PPARγ. Adult male and female Wistar rats underwent contextual fear conditioning, followed by THC (0.002 mg/kg, i.p.) or vehicle administration immediately after memory retrieval. Microglial involvement was assessed using immunofluorescence and pharmacological and chemogenetic inhibition in DH. The role of CB1 and PPARγ receptors was assessed via intra-DH selective antagonist infusion. THC impaired reconsolidation in males and females. In males, fear memory retrieval increased microglial engagement in the DH CA1 subfield, which THC further enhanced. Pharmacological and chemogenetic inhibition of microglia, as well as selective CB1 and PPARγ antagonism, blocked THC’s effects in males. In females, THC-induced reconsolidation blockade was cycle-dependent, occurring at estrus and diestrus but not at proestrus, and was mediated exclusively through CB1 activation. These findings identified sex-specific neuroimmune pathways mediating THC’s reconsolidation impairment, offering a mechanistic basis for novel sex-tailored therapeutic opportunities.

**Figure:**
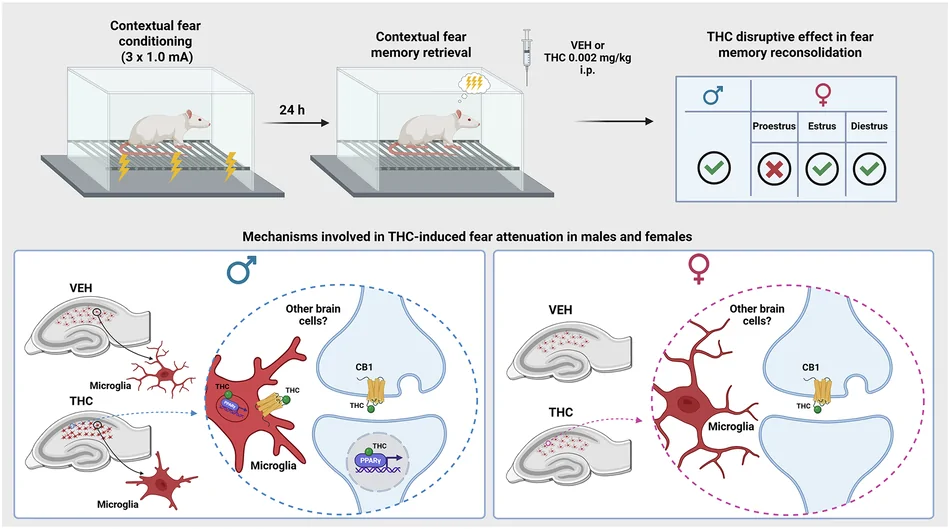

## Introduction

Targeting reconsolidation, a retrieval-dependent restabilization phase that enables memory updating, has emerged as a therapeutic approach for mitigating posttraumatic stress disorder (PTSD) symptoms [1–3]. PTSD is a debilitating, underdiagnosed, and undertreated psychiatric condition triggered by life-threatening events and marked by persistent, intrusive, traumatic memories [4] with higher prevalence in women. Women also have higher comorbidity and lower recovery rates than men [4, 5]. The extent to which this sex disparity stems from differences in experiential versus intrinsic biological factors remains unclear.

Growing evidence suggests that neuroimmune signaling plays a critical role in PTSD maintenance [6–9]. Microglia, the primary innate immune cells in the central nervous system, have increasingly recognized roles in regulating brain functions, including fear memory processing [10–12]. In rodent models, stress-induced microglial engagement contributes to aberrant phenotypes that mimic stress-related psychopathologies, including PTSD [13–17], which are modulated by sex [18–22]. For instance, males show a more robust microglial recruitment and a pro-inflammatory phenotype in response to neuroimmune stimuli than females [23–25]. Importantly, microglial depletion also facilitates fear memory extinction in male, but not female, PTSD rodent models [26].

Microglia exhibit increased activity in the dorsal hippocampus (DH) and prelimbic (PL) region of the medial prefrontal cortex (mPFC) following fear conditioning and retrieval, as evidenced by changes in cell number and morphology [12, 27, 28]. Notably, microglial engagement in the DH contributes to the destabilization of fear memory [29], a critical phase in the reconsolidation process.

Microglia cells also mediate the interplay between cannabinoids and neuroimmune mechanisms in stress-related disorders [6]. Activation of cannabinoid receptor type 1 (CB1) by synthetic ligands reduces microglial activity and attenuates stress-induced behavioral alterations [30], and the absence of CB1 prevents the stress-induced increase in microglial activity in the rat hippocampus [31]. Δ⁹-tetrahydrocannabinol (THC), a partial CB1 agonist, prevents behavioral impairments induced by lipopolysaccharide (LPS)-mediated neuroinflammation [32]. THC also activates peroxisome proliferator-activated receptor gamma (PPARγ), a nuclear receptor with well-established anti-inflammatory and neuroprotective actions [33–38]. Through this mechanism, THC regulates neuroinflammation, macrophage differentiation, and microglial polarization [39].

Previously, we showed that THC disrupts fear memory reconsolidation in male rats through mechanisms involving CB1 activation in the DH and PL cortex [40, 41]. In females, THC produces biphasic effects on anxiety-like behaviors at low doses that are ineffective in males [42]. We also found that proestrus females are insensitive to the effects of the fatty acid amide hydrolase (FAAH) inhibitor URB597 and the monoacylglycerol lipase (MAGL) inhibitor MJN110 on anxiety-like behavior [43]. Whether THC elicits an impairment of fear memory reconsolidation through sex-specific mechanisms remains unclear.

Hereinafter, we examined whether THC-induced impairment in reconsolidation is associated with a sex-dependent microglial recruitment and if this effect is mediated by CB1 or PPARγ in the DH using immunofluorescence and pharmacological and chemogenetic inhibition. Our results demonstrate that THC disrupts fear memory reconsolidation through both CB1- and PPARγ-dependent mechanisms involving microglial engagement in males, whereas in females this effect is mediated exclusively by CB1 signaling. These findings reveal a novel sex-specific neuroimmune pathway mediating THC’s effects on reconsolidation, providing a mechanistic framework for the development of more effective and personalized, sex-tailored therapeutic interventions for PTSD.

## Materials and methods

### Animals

Three-month-old adult Wistar rats (male and female, total n: 349) were obtained from the Biological Sciences Sector at the Federal University of Paraná. Rats were housed in groups of three or four per polycarbonate cage (48 × 37 × 21 cm), with *ad libitum* access to food and water, maintained at 22 ± 2 °C on a 12-h light-dark cycle (lights on at 7:00 a.m.). All procedures were approved by the Local Ethics Committee (authorization numbers 1526 and 1526 A) and adhered to Brazilian legislation and the Essential 10 ARRIVE 2.0 guidelines [44].

### Drugs

THC (AuraPharma, Uruguay; 0.002 mg/kg, i.p.), minocycline (MINO; microglia inhibitor; Sigma-Aldrich, USA; 10 μg/0.5 μL, intra-DH infusion), DREADD agonist 21 (C21; Sigma-Aldrich, USA; 1 mg/kg, i.p.), AM251 (CB1 receptor antagonist; Tocris, EUA; 0.5 nmol/0.5 μL, intra-DH infusion), and GW9662 (PPARγ antagonist; Sigma, EUA; 32 pmol/0.5 μL, intra-DH infusion) were used. THC and AM251 were dissolved in saline (0.9% NaCl; pH 7.04) with 5% Tween^©^ 80; GW9662 and MINO were dissolved in saline with 3% DMSO, and C21 was dissolved in saline. Each vehicle (VEH) solution served as a control for its respective drug. All solutions were prepared fresh before use. The doses were based on previously published studies [41, 45–47].

### Contextual fear conditioning

Fear conditioning was conducted as previously described [41], using a rectangular chamber (Context A; 35 × 20 × 30 cm, 70 lx) with aluminum side walls, a Plexiglass front, and a shock-grid floor connected to a shock generator (Insight, Brazil). During familiarization, rats explored Context A for 3 min. In the conditioning session, 24 h later, they received three 1.0 mA, 3-s foot shocks (with 30-s intervals before, between, and after), serving as the unconditioned stimulus (US). Memory retrieval was assessed the following day by re-exposing the rats to Context A for 3 min without presenting the US. Immediately after, rats were randomly assigned to treatment groups. The effects were evaluated 24 h later by re-exposing the rats to Context A for 3 min (Test A). Freezing behavior was recorded by a trained observer blinded to group assignments and served as an index of fear memory [48].

### Novel Object Recognition (NOR) task

The training session, conducted in a black quadrangular arena, consisted of a 10-min exposure to two identical objects (A and A). After 24 h, the recognition memory test was conducted for 10 min by replacing one of the familiar objects with a novel object (B). Object exploration times were recorded and used to calculate the discrimination index (DI). Detailed information is provided in the Supplementary Material.

### Estrous cycle determination

After memory retrieval, the estrous cycle phase was determined by collecting vaginal secretions using a micropipette tip containing 30 μL of saline. The samples were analyzed under a light microscope at 10x magnification to identify the phases (proestrus, estrus, and diestrus) based on the proportions of leukocytes, nucleated, and cornified cells [49].

### Analysis of THC and its metabolites

Male and female naive rats received THC (0.002 mg/kg i.p.). After 30 or 60 min, the animals were decapitated, the blood (1.5 mL) and the DH were collected. The samples were prepared according to ref. [50]. THC, 11-hydroxy-THC (11-OH-THC), and 11-nor-9-carboxy-THC (THC-COOH) were analyzed by high-performance liquid chromatography coupled to tandem mass spectrometry (HPLC-MS/MS) according to ref. [51]. Detailed information is provided in the Supplementary Material.

### Immunofluorescence

Fluorescence intensity of ionized calcium-binding adapter molecule 1 (Iba-1) was analyzed 90 min after treatment. Coronal brain sections (40 µm) were collected with a cryostat (Leica, Germany) at the levels of PL cortex and DH {AP from bregma: +3.8 mm to +2.2 mm and −2.4 mm to −4.6 mm [52]}. Images obtained at 20x were analyzed from both the PL cortex and the CA1 subfield of the DH (DH CA1). Detailed information is provided in the Supplementary Material.

### Stereotaxic surgery

Deeply anesthetized rats were positioned in a stereotaxic frame (David Kopf, USA). Using the following coordinates: AP = −3.8 mm for males and −3.2 mm for females, ML = ± 2.5 mm, DV = −1.8 mm for cannula implantation, and −3.3 mm for viral infusion, we targeted the DH CA1 bilaterally [52]. A representative histological image of the infusion site is shown in Supplementary Fig. S1. Detailed information is provided in the Supplementary Material.

### Viral infusion and chemogenetic inhibition

AAV1-CD68-hM4Di-mCherry (Addgene, USA) was infused to induce hM4Di expression in microglia in the DH CA1. The virus at a titer of 3.3 × 10^12^ vg/mL was delivered in a 1.0 μL/side volume. The experiments started 21 days post-surgery for viral expression. Detailed information is provided in the Supplementary Material.

### Statistical analysis

After testing for normality of the data using the Kolmogorov–Smirnov and Shapiro–Wilk tests, statistical analyses were performed using unpaired Student’s *t* tests, one-way, repeated-measures, or two-way repeated-measures ANOVA, as appropriate. The Newman–Keuls test was used for *post-hoc* comparisons (*p* < 0.05). Only F values from interactions were represented when they reached statistical significance. The partial eta-squared (η^2^) or Cohen’s *d* was calculated to estimate the effect size (η^2^ ≥ 0.14 and *d* ≥ 0.8 large effect [53]). Statistic 12 (StatSoft, USA) was used for statistical analysis, and GraphPad Prism 8 (GraphPad Prism, USA) was used for graphing. Sample sizes were determined using G-Power (HHU: Universität Düsseldorf, Germany), with 6–8 rats per group for immunofluorescence and 8–10 for behavioral tests, based on large expected effects [40, 41]. Although groups were designed to be equal, some were unequal due to experimental losses (e.g., mistargeted infusions or tissue damage).

## Results

### THC disrupts fear memory reconsolidation in males, and in estrus and diestrus females

**Fig. 1:**
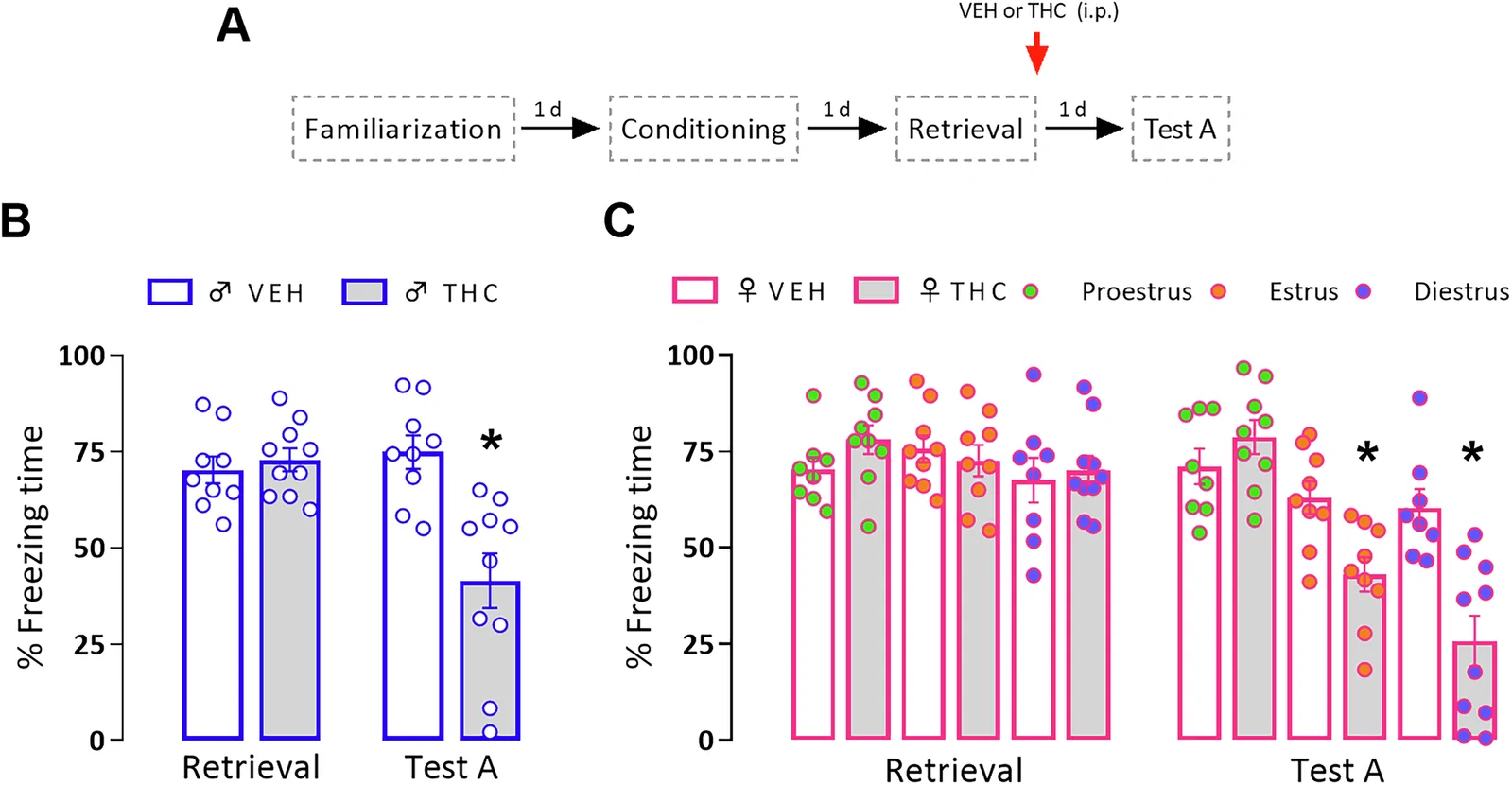
Effects of THC 0.002 treatment on fear memory reconsolidation in males and females. **A** Experimental design. **B** THC decreased the freezing time in Test A, suggesting an impairment in fear memory reconsolidation in males. n per group: VEH: 9, THC: 10. **C** In females, THC decreased the freezing time in Test A during estrus and diestrus phases, but not during proestrus, indicating that the effect of THC on reconsolidation depends on the estrous cycle phase. n per group: Proestrus-VEH: 8, Proestrus-THC: 9, Estrus-VEH: 9, Estrus-THC: 9, Diestrus-VEH: 8, Diestrus-THC: 10. Results are expressed as mean ± SEM and individual values. The * indicates *p* < 0.05 compared with the session control group.

The effect of THC on reconsolidation has been previously studied only in males [40, 41]. Here, we examined the effects of an ultra-low dose of THC administered immediately after retrieval in males and in naturally cycling females. In males, a significant treatment × context reexposure interaction was found [F_(1,17)_ = 15.7, *p* = 0.001; η² = 0.48; *n* = 9–10/group]. Treatment with THC (0.002 mg/kg, i.p.) significantly reduced the percentage of freezing time in Test A compared to controls (*p* = 0.0003; Fig. 1B), confirming an impairment in fear memory reconsolidation.

In females, a significant treatment × estrous cycle × context reexposure interaction was found [F_(2,47)_ = 8.2, *p* = 0.001; η² = 0.26; *n* = 8–10/group]. THC (0.002 mg/kg) reduced the percentage of freezing behavior in Test A compared to controls in estrus (*p* = 0.012) and diestrus (*p* = 0.0001) but failed to do so in proestrus (*p* = 0.94), supporting a cycle phase-dependent impairment in fear memory reconsolidation (Fig. 1C). Based on these findings, subsequent behavioral experiments were conducted exclusively in female rats in estrus and diestrus. Importantly, THC administration after fear memory retrieval did not affect NOR memory in either sex, since no differences were observed between groups in the DI of males [t_16_ = 0.77; *p* = 0.45; *d* = 0.36; *n* = 9/group] and females [t_14_ = 0.91; *p* = 0.38; *d* = 0.45; *n* = 8/group] (Supplementary Fig. S2).

Importantly, qualitative LC-MS/MS analyses revealed that the active THC metabolite, 11-OH-THC, was detected in blood and in the DH of both male and female rats at 30 and 60 min following THC administration. In contrast, parent THC was not detectable at these time points, consistent with the rapid metabolism of THC to its downstream metabolites [54] (Supplementary Figs. S3, S4).

### THC enhances microglial recruitment during reconsolidation in the DH CA1 of male rats

**Fig. 2:**
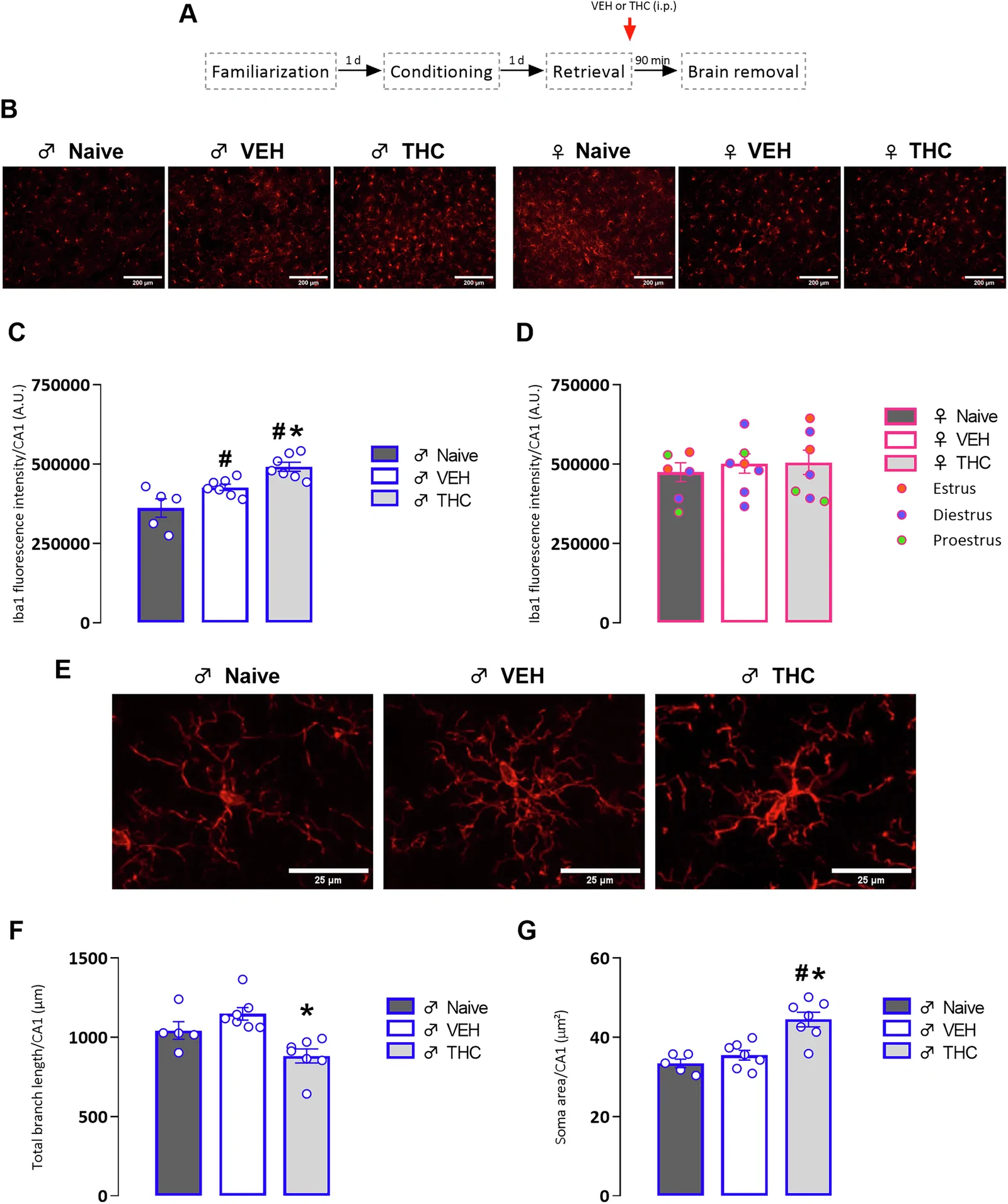
Effects of THC on microglial engagement during fear memory reconsolidation in the DH CA1 subfield. **A** Experimental design. **B** Representative photomicrographs of Iba-1+ cells at 20× magnification. Scale bar = 200 μm. **C** In males, THC treatment increased Iba-1 fluorescence intensity in the DH CA1 subfield. Furthermore, the VEH and THC groups showed higher Iba-1 fluorescence intensity than Naive rats, suggesting that fear conditioning induces microglial recruitment in the DH. THC further enhanced this recruitment in males. *n* per group: Naive: 5, VEH: 7, THC: 7. **D** In females, no difference in the Iba-1 fluorescence intensity in the DH CA1 subfield was detected. *n* per group: Naive: 6, VEH: 7, THC: 7. **E** Representative micrographs of Iba-1+ cells at 60× magnification. Scale bar = 25 μm. **F** THC treatment decreased the total branch length of microglia cells in the DH CA1 subfield of males. **G** THC treatment increased microglial soma area in the DH CA1 subfield in males. Results are expressed as mean ± SEM and individual values. The * indicates *p* < 0.05 compared with the VEH group. The # indicates *p* < 0.05 com*p*ared to the naive group.

To study microglia’s involvement in THC-induced reconsolidation impairment, we evaluated the fluorescence intensity of Iba-1 – a microglia-specific marker – in the DH CA1 and PL cortex in both sexes 90 min after memory retrieval. In the DH CA1, a significant effect was found in males [F_(2,16)_ = 13.5; *p* = 0.0004; η^2^ = 0.63; *n* = 5–7/group]. Treatment with THC (0.002 mg/kg) significantly increased Iba-1 fluorescence intensity compared to VEH-treated male rats (*p* = 0.032; Fig. 2C). Importantly, both VEH- and THC-treated groups exhibited enhanced Iba-1 fluorescence intensity compared to naive rats (*p*_*naivexVEH*_ = 0.048 and *p*_naivexTHC_ = 0.0003).

In male DH CA1, a significant difference among groups was found for total branch length [F_(2,16)_ = 9.7; *p* = 0.002; η^2^ = 0.55] and soma area [F_(2,16)_ = 15.3; *p* = 0.0002; η^2^ = 0.66]. THC treatment significantly reduced the total branch length (*p* = 0.002) and increased the soma area (*p* = 0.002) of microglia in the DH CA1 compared to VEH-treated rats (Fig. 2F, G). This suggests that THC enhances microglial engagement by altering its morphology during fear memory reconsolidation in the DH CA1 in male rats.

In female rats, no significant differences in Iba-1 fluorescence intensity were observed in the DH CA1 among groups [F_(2,17)_ = 0.23; *p* = 0.79; η^2^ = 0.03; *n* = 6–7/group], indicating an absence of microglial recruitment (Fig. 2D). However, naive females showed significantly higher Iba-1 fluorescence intensity in the DH CA1 than naive males, suggesting a sex difference in microglial recruitment [t_9_ = 2.68; *p* = 0.025; *d* = 1.6].

In the PL cortex, no significant differences in Iba-1 fluorescence intensity were observed among groups in either males [F_(2,16)_ = 0.60; *p* = 0.56; η^2^ = 0.07], or females [F_(2,17)_ = 0.67; *p* = 0.52; η^2^ = 0.07], supporting the absence of microglial recruitment in this brain area during fear memory reconsolidation and following THC treatment (Supplementary Fig. S5). No difference in Iba-1 fluorescence intensity in PL between both naive females and males was detected [t_9_ = 0.23; *p* = 0.825; *d* = 0.13].

### Microglia mediate THC-induced impairment of fear memory reconsolidation in the DH in males but not in females

To assess microglial involvement in the THC-induced impairment of fear memory reconsolidation and given the increased microglial recruitment observed in the DH CA1 of THC-treated males, we next examined whether pharmacological or chemogenetic inactivation of microglia within the DH would prevent this effect on fear responses. Although Iba-1 immunofluorescence did not reveal significant changes in female microglia, inclusion of female cohorts under identical experimental conditions in pharmacological and chemogenetic approaches remains crucial to determine whether THC engages distinct cellular mechanisms across sexes. Also, it allows us to determine whether, even in the absence of increased microglial recruitment, microglia may nonetheless contribute to reconsolidation blockade. Thus, these complementary approaches are pivotal in reinforcing the notion that microglia are engaged by THC in males, while reaffirming that, in females, reconsolidation impairment occurs through microglia-independent mechanisms.

Also, Iba-1 immunofluorescence was evaluated 90 min after memory retrieval. In pharmacological experiments, MINO was administered intra-DH immediately after retrieval, followed by i.p. injection of THC. C21 was administered systemically 30 min before retrieval and THC immediately after retrieval. Previous studies have demonstrated that the microglial inhibitory action of MINO persists for over six hours, a period consistent with the active reconsolidation window (~6 h) during which memory traces remain labile and susceptible to pharmacological interference [55].

#### Pharmacological inhibition of microglia in the DH prevents the THC-induced impairment of reconsolidation only in male rats

**Fig. 3:**
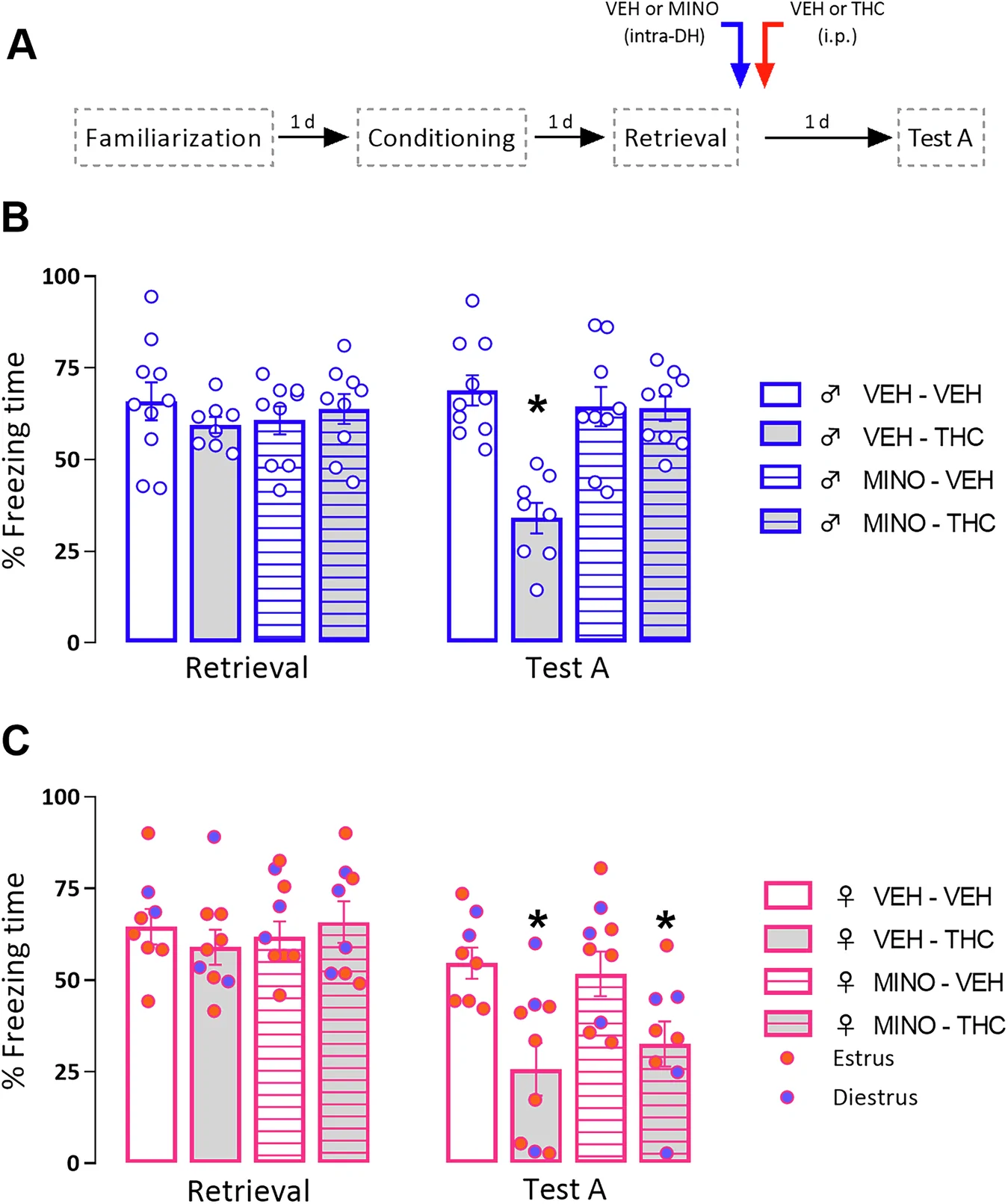
Effects of pharmacological inhibition of DH microglia on THC-induced effects. **A** Experimental design. **B** THC treatment in males decreased the freezing time in Test A compared to controls. This effect was prevented by pretreatment with MINO into the DH immediately after memory retrieval, just before THC i.p. treatment, suggesting that hippocampal microglial activity is required for the THC-induced effects on fear memory reconsolidation in males. n per group: VEH-VEH: 10, VEH-THC: 8, MINO-VEH: 9, MINO-THC: 9. **C** THC treatment in females decreased the percentage of freezing time in Test A compared to controls. This effect was not prevented by MINO, suggesting that the effect of THC on fear memory reconsolidation in females does not depend on hippocampal microglial activity. n per group: VEH-VEH: 8, VEH-THC: 9, MINO-VEH: 9, MINO-THC: 8. Results are expressed as mean ± SEM and individual values. The * indicates *p* < 0.05 compared with the session control group.

In males, a significant pretreatment × treatment × context reexposure interaction was found [F_(1,31)_ = 4.8; *p* = 0.037; η^2^ = 0.13; *n* = 8–10/group]. THC-induced reconsolidation impairment (*p* = 0.0002) was prevented in rats pretreated with the microglia inhibitor, MINO (*p* = 0.90), indicating the involvement of microglia in THC’s effects on reconsolidation in male rats (Fig. 3B).

As expected, females showed no significant pretreatment × treatment × context reexposure interaction [F_(1,30)_ = 0.03; *p* = 0.86; η^2^ = 0.001; *n* = 8–9/group] or pretreatment effect [F_(1,30)_ = 0.35; *p* = 0.56; η^2^ = 0.01], but there were significant effects of treatment [F_(1,30)_ = 9.0; *p* = 0.005; η^2^ = 0.23] and context reexposure [F_(1,30)_ = 58.4; *p* < 0.00001; η^2^ = 0.66]. THC-induced reconsolidation impairment (*p* = 0.001) was not prevented by pretreatment with MINO (*p* = 0.009), further supporting that the THC-induced impairment in fear memory reconsolidation is microglia-independent in females (Fig. 3C).

#### Activation of hM4Di in the DH abolishes the THC-induced impairment in reconsolidation only in male rats

**Fig. 4:**
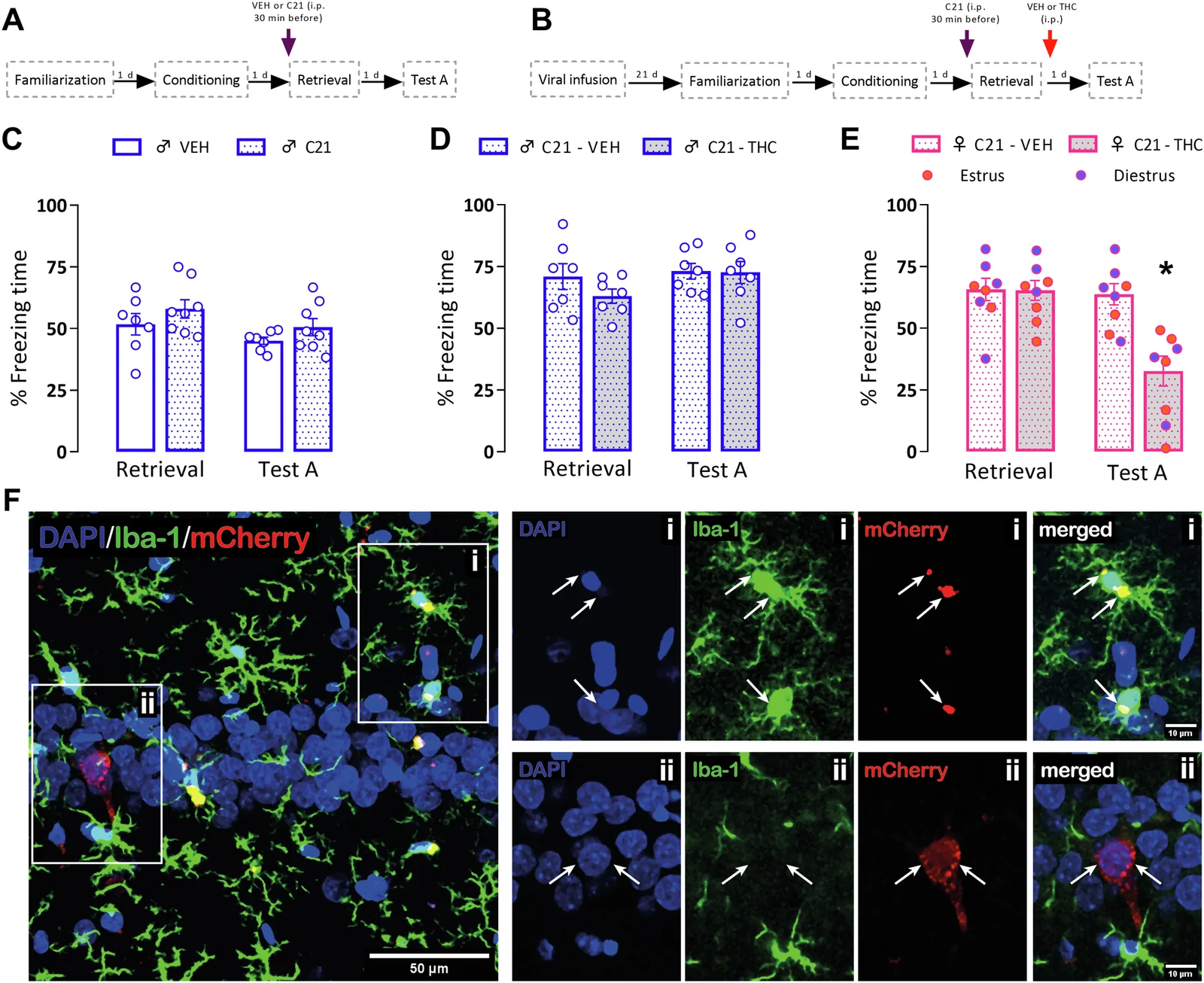
Effects of hM4Di activation in the DH on THC-induced modulation of fear. **A** Experimental design of data reported in (**C**). **B** Experimental design of data depicted in (**D**) and (**E**). **C** In non-transfected male rats, C21 (1 mg/kg, i.p.) administered 30 min before retrieval did not change freezing behavior during retrieval or Test A (VEH: *N* = 7, C21: *N* = 9). **D:** In males, hM4Di activation in the DH CA1 with C21 (1 mg/kg, i.p.) administered 30 min before retrieval prevented the pharmacological effect of THC in Test A, confirming that THC’s effect on fear memory reconsolidation in males depends on hippocampal microglial activity (C21-VEH: *N* = 7, C21-THC: *N* = 7). **E** In females, hM4Di activation in the DH CA1 did not prevent THC-induced reduction in freezing in Test A, further suggesting that the effect of THC on fear memory reconsolidation in females is independent of hippocampal microglial activity (C21-VEH: *N* = 8, C21-THC: *N* = 8). **F** Confocal images of the DH CA1 subfield showing mCherry and Iba-1 expression. White boxes indicate regions shown at higher magnification with separate channels (DAPI, Iba-1, mCherry) and merged views. Scale bar = 50 μm. In (i), mCherry signal co-localizes with Iba-1, identifying microglial cells. In contrast, (ii) shows mCherry+ cells that do not express Iba-1, indicating a non-microglial subset of cells with neuron-like morphology. Scale bar = 10 μm. Results are expressed as mean ± SEM and individual values. The * indicates *p* < 0.05 compared with the session control group.

We employed a chemogenetic approach to inactivate microglia by expressing hM4Di under the CD68 promoter in the DH CA1 and administering C21 (1 mg/kg, i.p.), a selective DREADD agonist that dampens cellular activity through hM4Di signaling, to confirm their involvement in the THC-induced effects on reconsolidation. In non-transfected male rats, there were no significant effects of treatment × context reexposure interaction [F_(1,14)_ = 0.26; *p* = 0.61; η^2^ = 0.018; *n* = 7–9/group] and no significant effects of treatment [F_(1,14)_ = 2.8; *p* = 0.12; η^2^ = 0.16]. C21 treatment 30 min before memory retrieval does not affect freezing behavior (Fig. 4C). An effect of context reexposure [F_(1,14)_ = 11.7, *p* = 0.004; η^2^ = 0.45] was observed. In males, there were no significant treatment × context reexposure interaction [F_(1,14)_ = 0.09; *p* = 0.77; η^2^ = 0.006; *n* = 7/group], effects of treatment [F_(1,14)_ = 0.33; *p* = 0.58; η^2^ = 0.02] or context reexposure [F_(1,14)_ = 4.6; *p* = 0.05; η^2^ = 0.25]. THC (0.002 mg/kg) failed to reduce freezing time in Test A in rats pretreated with C21 (Fig. 4D). Figure 4F confirms the co-expression of mCherry in Iba-1+ cells in the DH CA1. However, we also observed off-target mCherry expression in a subset of non-Iba-1+ cells with neuronal-like morphology. These findings indicate that chemogenetic inhibition of microglia in the DH CA1 abolished THC’s effects on fear in male rats. However, we cannot rule out a potential contribution of CA1 pyramidal neurons to these effects.

In contrast, in females, a significant treatment × context reexposure interaction was observed [F_(1,14)_ = 13.7; *p* = 0.002; η² = 0.49; *n* = 8/group]. Pretreatment with C21 (*p* = 0.0002; Fig. 4E) failed to block THC (0.002 mg/kg) impairing effects on fear memory reconsolidation.

### THC affects fear memory reconsolidation via CB1 receptors in both sexes but involves PPARγ only in male rats

**Fig. 5:**
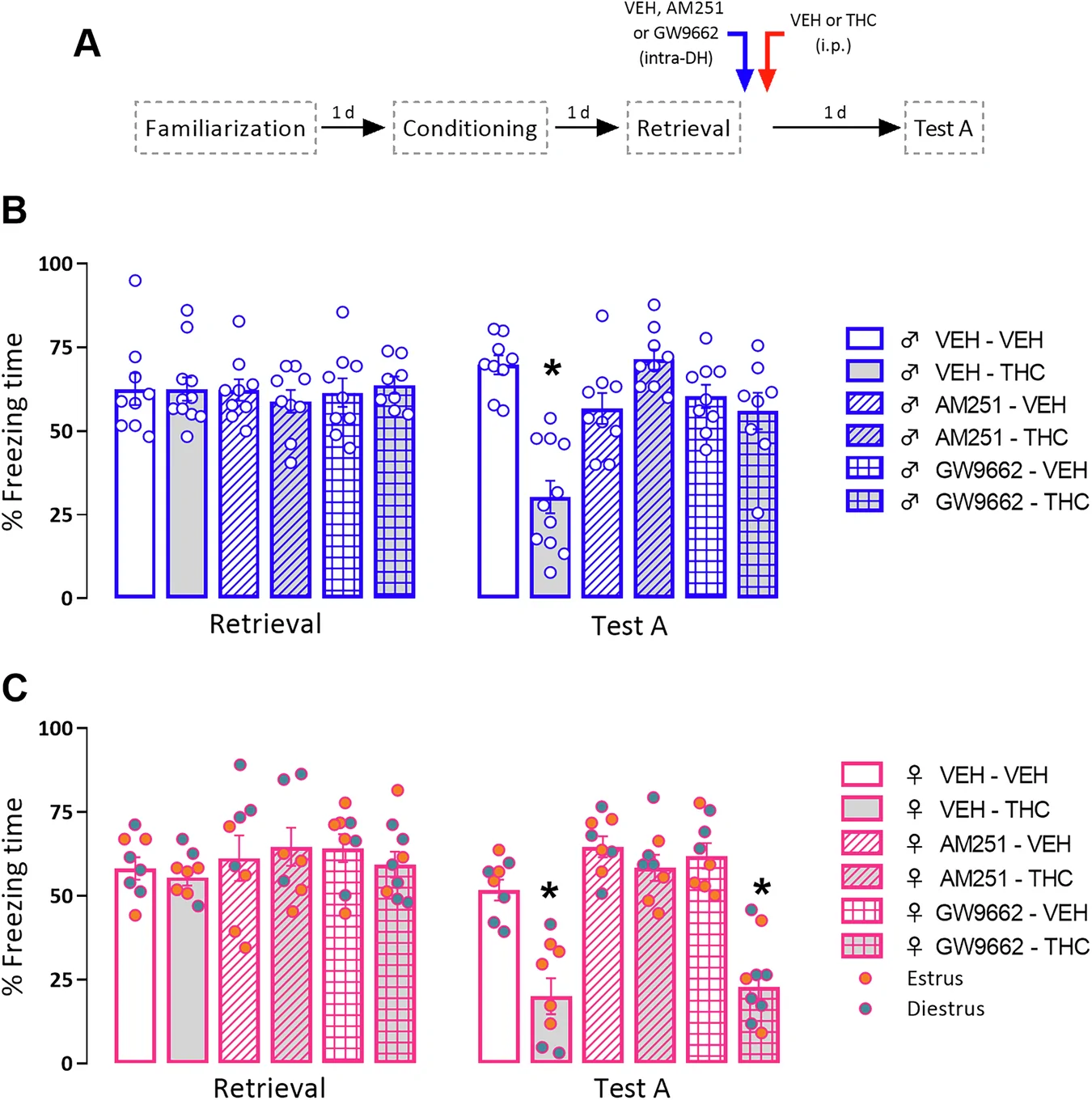
Effects of CB1 and PPARγ receptors antagonism in the DH on the effects of THC. **A** Experimental design. **B** Treatment with THC in males decreased the percentage of freezing in Test A compared to the control, and this effect was prevented by pretreatment with AM251 or GW9662 infused into the DH immediately after memory retrieval, suggesting that the effect of THC on fear memory reconsolidation in males depends on CB1 and PPARγ receptors in the DH. n per group: VEH-VEH: 9, VEH-THC: 11, AM251-VEH: 9, AM251-THC: 9, GW9662-VEH: 9, GW9662-THC: 8. **C** THC treatment in females decreased the freezing percentage in Test A compared to the control, but this effect was only prevented by AM251, not by GW9662, indicating that the effect of THC on fear memory reconsolidation in females depends on CB1, but not on PPARγ receptors in the DH. n per group: VEH-VEH: 8, VEH-THC: 8, AM251-VEH: 8, AM251-THC: 8, GW9662-VEH: 8, GW9662-THC: 9. Results are expressed as mean ± SEM and individual values. The * indicates *p* < 0.05 compared with the session control group.

Both CB1 and PPARγ receptors regulate microglial engagement and are also targets of THC for its pharmacological effects. Here, we investigated if THC’s reconsolidation impairing effects occur through CB1 or PPARγ, specifically expressed in the DH, and whether there are any sex-related differences in the action of THC through these receptors. In males, a significant pretreatment × treatment × context reexposure interaction was found [F_(2,50)_ = 18.3; *p* < 0.00001; η^2^ = 0.42; *n* = 8–10/group]. THC-induced reconsolidation impairment (*p* = 0.0002) was prevented by intra-DH pretreatment with the CB1 antagonist, AM251 (*p* = 0.79), but also by intra-DH pretreatment with the selective PPARγ antagonist, GW9662 (*p* = 0.75). These results suggest that both CB1 and PPARγ receptors participate in THC-induced impairment of fear memory reconsolidation in male rats (Fig. 5B).

In females, a significant pretreatment × treatment × context reexposure interaction was found [F_(2,43)_ = 3.3; *p* = 0.045; η² = 0.14; *n* = 8–9/group]. THC-induced reconsolidation impairment (*p* = 0.0001) was prevented by pretreatment with AM251 (*p* = 0.70). In contrast, GW9662 pretreatment failed to block the THC effect (*p* = 0.0001). The results suggest that, in comparison to males, THC-induced impairment of memory reconsolidation in female rats is mediated exclusively through CB1 receptor activation (Fig. 5C).

## Discussion

The present study provides the first evidence that THC, at an ultra-low dose, disrupts fear memory reconsolidation through a mechanism involving microglia function and endocannabinoid signaling in the rat DH. This effect was clearly dependent on sex and the estrous cycle. In males, but not in females, this disruption required microglial recruitment, as pharmacological inhibition or chemogenetic silencing of microglia completely abolished THC’s effect on fear memory. Moreover, receptor-specific contributions mediating these pharmacological effects were identified. In females, CB1 signaling predominantly mediated THC-induced effects on reconsolidation, whereas in males, both CB1 and PPARγ were involved. These results uncover a novel sex-specific neuroimmune–endocannabinoid interaction in the regulation of fear memory, underscoring the importance of microglial activity in determining the outcome of cannabinoid-based interventions.

### THC’s impairment of reconsolidation across sex and estrous cycle

The administration of THC immediately after fear memory retrieval blocked the fear memory reconsolidation in rats. Prior studies have largely been limited to male rodents and have reported that the same dose used in our study (0.002 mg/kg), administered before retrieval, or higher doses (0.3–10 mg/kg) administered after retrieval, impaired fear memory reconsolidation [40, 41]. However, there is also contrasting evidence reporting no effect of a high THC dose (50 mg/kg) on the reconsolidation process [56]. Our findings extend this literature by showing that a low-dose THC also impairs reconsolidation in females, in a cycle-specific way, with efficacy restricted only to estrus and diestrus, when ovarian steroid levels (estradiol and progesterone) are low [57], but not during proestrus, when sex steroid levels are high [57]. These hormonal fluctuations across the estrous cycle may underlie behavioral phenotypes observed in response to THC administration. Elevated levels of anandamide and 2-AG are associated with alterations in anxiety-like behavior during non-proestrus phases [43]. Also, estradiol modulates CB1 receptor density and affinity in brain regions underlying memory destabilization and reconsolidation, including the DH [58, 59]. Furthermore, elevated estradiol and progesterone and their GABAergic metabolites, with a recognized role in PTSD pathophysiology [60, 61], may buffer against THC’s pharmacological effects, which may account for the absence of THC’s effect during proestrus. Notably, the treatment with THC after fear memory retrieval did not affect the subsequent learning of a neutral memory, as assessed in the NOR test, underscoring the specificity of THC’s effects for fear-related reconsolidation processes.

### Microglia modulation of THC’s effects on reconsolidation is male sex-specific

Ninety minutes post-retrieval, THC treatment significantly increased Iba-1 fluorescence intensity, reduced total branch length, and enlarged soma area of microglia in the DH CA1 of male rats, suggesting enhanced microglial engagement and morphological remodeling compared to controls during the reconsolidation window. This result aligns with Chaaya et al. [12], who reported an increase in Iba-1+ cells in the DH CA1, 90 min after contextual fear conditioning, and with studies showing enhanced microglial engagement in the male DH from 1–32 days after fear conditioning [62–66]. Neuronal activation during fear conditioning, beyond the stressor itself, may contribute to this microglial response, reflecting the mutual interactions between neurons and microglia in memory maintenance [67]. No significant changes were detected in microglia in the PL cortex after retrieval, reflecting region-specific and temporal dynamic differences. For example, Chaaya et al. [27] reported an increased number of Iba-1+ cells, without changes in morphology, in the PL cortex, 12 days after fear conditioning. Unlike males, females showed no changes in Iba-1 expression both in the DH CA1 and PL cortex following THC administration. This finding is consistent with previous studies showing the lack of microglial modulation in females subjected to single prolonged stress (SPS) [26] or unpredictable chronic stress [68]. However, naive females showed higher Iba-1 fluorescence intensity than males in the DH CA1, but not in the PL, suggesting that females exhibit greater basal microglial recruitment, although it does not seem to modulate conditioned fear behavior. Indeed, there is evidence that females may have more microglial cells in adulthood than males in the DH [69], although other studies have not shown a sex difference in this brain area [19, 70]. Collectively, these findings support low microglial responsiveness to reconsolidation processes, likely due to hormonal regulation, cellular types, such as astrocytes, alternative glial-neuronal interactions, or direct neuronal CB1 signaling, compensating for the lack of microglial engagement in females.

In males, THC’s effect was prevented by pharmacological inhibition of microglia through MINO administration intra-DH. Similar effects were observed when a high dose (20 mg/kg) of THC-induced impairment in cerebellar-dependent conditioned learning, and this effect was reversed by MINO pretreatment [71]. However, few investigations have addressed the role of microglia in reconsolidation, and none have examined sex-dependent effects. While evidence indicates that microglial suppression impairs memory reactivation [29], a critical step in reconsolidation, other studies assessing remote memory or repeated microglial inhibition have shown that this process does not affect fear memory retrieval or reconsolidation [66, 72]. MINO is a tetracycline widely used to suppress microglial activity and responsiveness [73]. It has off-target effects, including modulation of neurotransmitters and reduction of oxidative stress [74, 75]. Evidence shows that chemogenetic hM4Di activation under the CD68 promoter provides selective and reversible silencing of microglia [76], offering a more precise approach than conventional pharmacology.

Even though mCherry expression was not microglia-restricted, administration of C21 prevented THC-induced reconsolidation impairment only in males. This converging evidence from pharmacological and chemogenetic strategies strengthens the finding that microglia are mediators, though not necessarily exclusive, of THC’s reconsolidation effects in males, but not in females. However, this does not exclude the participation of other brain cell types, such as CA1 pyramidal neurons, in mediating the effects of hM4Di activation in males, suggesting a potential microglia-neuronal interaction. In females, MINO administration and chemogenetic inhibition failed to prevent THC’s effect, consistent with the absence of THC-induced changes in Iba-1 fluorescence intensity and further suggesting that microglial involvement in reconsolidation is sex-specific. These findings align with evidence that microglia may not be essential mediators of memory processes in females [23, 26, 77–79], at least not via THC-related pathways.

Compared with other DREADD agonists, such as CNO, C21 is not converted to clozapine, thereby reducing its potential off-target effects [80]. Consistent with this premise, in non-transfected rats, C21 did not alter freezing behavior. Accordingly, the same dose of C21 used herein did not change locomotion, exploratory behavior, and anxiety-like behaviors in non-transduced animals [81], or freezing behavior in AAV-CaMKIIα-EGFP animals (neuronal DREADD controls) [47].

Previous studies have shown that AAV1-CD68-hM4Di-mCherry exhibits preferential expression in microglia. For instance, a selective microglial infection was observed in the locus coeruleus of rats and in the mouse cortex [82, 83]. Evidence suggests that the AAV serotypes’ efficiency of infection differs across different brain areas and that AAV1 infects the DH with affinity for neurons and glial cells, including microglia [84]. Then, it is possible that the off-targets observed in our study may result from variations in cellular composition and microenvironment among brain areas.

In general, microglial engagement has been associated with the release of inflammatory mediators and synaptic remodeling mechanisms that modulate neuronal activity, suppressing hyperexcitability and neurotransmitter release, and promoting dendritic spine pruning [85–87]. Such effects were observed in stress models, including fear conditioning [59], acute restraint stress [88], and unpredictable chronic stress [65]. Microglia-mediated pruning has been linked to memory formation and maintenance in the CA1 [59, 89]. Thus, THC-induced microglial engagement during reconsolidation in males may modulate neuronal circuits storing the memory by pruning and affecting neurotransmission, potentially leading to reduced fear responses. In contrast, in females, microglial failure to modulate THC-induced reconsolidation impairment highlights a functional divergence in neuroimmune contribution across sexes with implications for refining therapeutic interventions.

### Receptor involvement in THC’s effects on reconsolidation

In males, THC’s effect on reconsolidation was prevented by administration of CB1 or PPARγ antagonists infused into the DH, while in females, only CB1 antagonism was effective. Previous studies have shown that both CBD and THC (0.3 mg/kg) disrupt reconsolidation in males via CB1 expressed in the mPFC [40, 90]. In females, the CBD-impairing effect also depended on CB1 in the DH [91], suggesting that this receptor is a key mediator in cannabinoid-induced modulation of fear memory in both sexes. Consistent with pharmacokinetic considerations indicating rapid THC metabolism, the presence of the active THC metabolite, 11-OH-THC, in the DH, supports the participation of CB1, given that 11-OH-THC retains significant activity at this receptor [54, 92]

In addition to the CB1 receptor, THC’s pro-cognitive effects in LPS-treated mice also required PPARγ [32], as did CBD’s impairment of fear memory consolidation [46]. Given the lack of evidence supporting PPARγ affinity for 11-OH-THC, PPARγ involvement in THC’s pharmacological effects may arise from direct activation by THC. Alternatively, PPARγ engagement may occur indirectly by inhibiting fatty acid–binding proteins (FABPs), thereby elevating anandamide levels and consequently activating both CB1 and PPARγ receptors [93, 94]. Furthermore, while systemic and brain THC concentrations can peak between 15 and 30 min after i.p. administration and decline rapidly thereafter [54], even a brief window of THC exposure at its receptors may be sufficient to initiate downstream signaling cascades. Transient receptor engagement can drive long-term transcriptional programs that, in turn, influence intracellular signaling pathways, extending the signal beyond the period when parent THC is readily quantifiable. For instance, activation of PPARγ engages both transcription-dependent and rapid signaling mechanisms. These include the regulation of genes involved in mitochondrial function, inflammation, synaptic plasticity, and neurosteroid biosynthesis, as well as the repression of pro-inflammatory transcription factors, such as NF-κB [95–99]. In parallel, PPARγ activation can also trigger non-genomic signaling through kinase pathways, including ERK/MAPK, which modulate synaptic plasticity, learning, memory, and fear conditioning [100–106]. Through these combined genomic and non-genomic routes, PPARγ signaling can propagate and sustain cellular responses that extend beyond the initial ligand exposure, ultimately shaping neuroinflammatory tone, neuroplastic adaptations, and fear-related behaviors.

Our data indicate a sex-specific role for PPARγ in reconsolidation. Evidence suggests that females may be less responsive to PPARγ agonists [107, 108]. Estradiol has been shown to downregulate peripheral PPARγ expression in models of allergy and diabetes [109, 110], a phenomenon that may also occur in the brain. Given PPARγ’s known anti-inflammatory and neuroprotective functions [34, 35], as well as its role in regulating macrophage and microglial polarization [36, 38, 111], an increase in PPARγ microglial expression under stress or pro-inflammatory conditions [38, 112] may enhance microglial responsiveness to THC’s effects in males. In contrast, lower PPARγ signaling, likely triggered by estradiol-mediated downregulation of PPARγ expression and a generally less pro-inflammatory microglial phenotype, may limit microglial involvement in response to THC, thereby resulting in a microglia-independent mechanism of reconsolidation modulation in females.

Although our data strongly suggest CB1 and PPARy involvement in microglial recruitment, we did not directly test whether antagonism to these receptors prevents THC-induced microglial recruitment. Future studies combining receptor antagonism with cellular readouts of microglial activity will be essential to clarify this causal link. Another limitation of our study is that it does not clarify whether THC acts primarily by activating CB1 and PPARγ expressed in neurons that, in turn, recruit microglia, or by directly targeting receptors expressed on microglia. For example, CB1 signaling on hippocampal GABAergic interneurons has been shown to influence microglial activity [113]. Moreover, the potential contributions of other brain cell types cannot be excluded. CB1 receptors are also present in astrocytes, where they regulate synaptic plasticity and behavior in a sex-specific manner [114]. Additionally, both CB1 and PPARγ are expressed in other glial populations, and phytocannabinoids may modulate their activity [115], potentially contributing to the effects observed in our study in females.

### Methodological considerations

Our data demonstrate the presence of the THC’s active metabolite, 11-OH-THC, in both blood and brain samples in male and female rats. However, methodological limitations such as the detection limit of the analytical technique (i.e., LS-MS/MS) used in this study and the selected sampling time points (30 and 60 min after THC administration) that conformed with the behavioral experiments of fear responses, may have contributed to the lack of detectable THC. The LC-MS method used to identify 11-OH-THC was qualitative, which limits conclusions about sex differences in pharmacokinetics and the relative abundance of this metabolite [50].

Potential off-target effects of MINO, including modulation of neurotransmitters and attenuation of oxidative stress, may also have influenced our results [74, 75]. To overcome this limitation, we further investigated microglial involvement using chemogenetic inhibition, which is regarded as more selective and refined than pharmacological interventions [116, 117]. However, we found that AAV1-CD68-hM4Di-mCherry infusion infected microglia and other brain cell subsets with neuron-like morphology; therefore, we cannot rule out a potential contribution of CA1 neuronal modulation to mediating THC’s pharmacological effect. CD68 is widely used as a microglial/macrophage marker; it is a lysosomal-associated protein whose expression reflects cellular phagocytic and degradative activity rather than strict lineage specificity [118, 119]. Indeed, its expression can be detected at low levels in non-myeloid cell types [120] under specific physiological or pathological conditions, including within the brain. Furthermore, in viral vector systems, promoter-driven expression may not fully recapitulate endogenous specificity, due to construct design and context-dependent regulation. For instance, in the DH, AAV1 has tropism for all cell types [84, 121], which may contribute to the off-target expression observed in our study.

## Conclusion

We showed that THC disrupts fear memory reconsolidation through sex-specific mechanisms. In males, the effect is mediated by microglial engagement in the DH CA1, though not necessarily exclusively, and involves CB1 and PPARγ signaling. In females, THC acts independently of microglia and PPARγ, relying solely on CB1 signaling. These findings highlight the importance of considering sex differences when developing cannabinoid-based treatments for fear memory-related disorders. Our findings support translating ultra-low-dose THC to target traumatic memory reconsolidation processes in PTSD. The efficacy of THC at a dose well below psychotomimetic thresholds could significantly minimize THC’s adverse effects. Moreover, sex-specific cellular mechanisms underlying THC’s action highlight the need for individualized approaches in PTSD treatment. Future human studies should investigate hormonal status, circulating inflammatory mediators, and microglial imaging biomarkers as potential predictors of the efficacy of cannabinoid-based therapies.

## Supplementary information

Supplementary material

## Data Availability

All data supporting this work are available from the corresponding author upon reasonable requests.
