# Supplementary material for: Sex-specific role of microglia in Δ9-tetrahydrocannabinol-induced disruption of fear memory reconsolidation
Source: Neuropsychopharmacology. 2026 May 9;51(11):2163–74. doi: 10.1038/s41386-026-02434-x (PMC13598131; doi:10.1038/s41386-026-02434-x)
Supplement: Supplementary file 1 [file 41386_2026_2434_MOESM1_ESM.docx]

1. **Supplementary methods**

**1.1 Immunofluorescence**

Ninety minutes after treatment, animals were deeply anesthetized and transcardially perfused with phosphate-buffered saline (PBS) and 4% paraformaldehyde (PFA). Brains were removed, post-fixed in 4% PFA for 24 h, and cryoprotected by immersion in a sucrose gradient (10%, 20%, and 30%; 24 h in each concentration). Tissues were frozen on dry ice, coronally sectioned at 40 μm using a cryostat (Leica, Germany), and processed for immunofluorescence, as described by Meyer et al. [1].

Briefly, free-floating sections were incubated in blocking solution [0.3% Triton X-100, BSA 1% in TBS, 3 h, room temperature (RT)] following incubation with polyclonal rabbit anti-ionized calcium-binding adapter molecule 1 (Iba-1) antibody (1:1000, Wako Chemicals USA, cat. no. 019-19741) overnight at 4 °C. The following day, sections were incubated for 2 h at RT with the respective donkey anti-rabbit secondary antibody (1:1000, Alexa Fluor^TM^ 594, Thermo Fisher Scientific, cat. no. A-21207, USA) and 4′,6-diamidino-2-phenylindole (DAPI, final concentration 0.025 µg/mL, Sigma Aldrich, cat. no. D9542). The sections were mounted with Fluoromount^TM^ (Sigma Aldrich, cat. no. F4680) on previously gelatin-coated slides.

For immunofluorescence analysis, images were acquired at 20x magnification using a fluorescence microscope (BX51 Olympus, Olympus Life Science, Japan). For each animal, at least three brain sections and two images per section were analyzed from both the PL cortex and the CA1 subfield of the DH (DH CA1), primarily from the stratum radiatum and lacunosum-moleculare. Fluorescence intensity was quantified using Fiji/ImageJ software. In brief, a threshold for positive staining was determined for each image, including all cell bodies and processes while excluding background signal. Data were processed by densitometric scanning of the thresholded areas, as described by Lisboa et al. [2].

When a significant difference in Iba-1 fluorescence intensity was detected, morphological analyses were performed. For these analyses, images were acquired at 60x magnification using confocal microscopy (Confocal A1R MP+, Nikon, Japan). Stitched Z-stack images were obtained and displayed as maximum intensity projections for analysis using Fiji/ImageJ software (NIH, USA). The soma area and total branch length were measured, and the results were expressed as the mean of 6–9 microglial cells analyzed per rat [[1]](https://www.zotero.org/google-docs/?KAOCNC). The projections were manually traced using the semiautomatic SNT plugin extractor of Fiji/ImageJ software.

**1.2 Novel object recognition (NOR) task**

Three days following Test A, male and female fear-conditioned rats were subjected to the NOR task. Animals were individually placed in a black quadrangular arena (60×60×30 cm) for 10 min per day over two consecutive days for habituation. The training session, conducted the following day, consisted of a 10-min exposure to two identical objects (A and A) positioned symmetrically along the arena's central horizontal axis. Twenty-four hours later, the recognition memory test was conducted by replacing one of the familiar objects with a novel object (B), and animals were allowed to explore for 10 minutes. Object exploration times were recorded and used to calculate the discrimination index (DI) using the formula: DI = (T_novel_ − T_familiar_) / (T_novel_ + T_familiar_), where T_novel_ and T_familiar_ represent the time spent exploring the novel and familiar objects, respectively. DI values approaching 1 indicate robust recognition memory, reflecting a strong preference for the novel object.

**1.3 Stereotaxic surgery**

Animals were deeply anesthetized with ketamine 75 mg/kg and xylazine 10 mg/kg and positioned in a stereotaxic frame (David Kopf, USA). They received a local anesthetic (3% lidocaine with vasoconstrictor adrenaline) at the incision site. Stainless-steel guide cannulas (9.0×0.7 mm) were implanted and fixed with acrylic resin and microscrews. To prevent blockage, we inserted a stylet into each cannula. Post-surgery, rats received Flunixin Meglumine (2.5 mg/kg; Banamine^®^, MSD Saude Animal, Brazil) *i.m.* and ibuprofen (0.2 mg/mL; Natulab, Brazil) in drinking water for 48 h. After 10 days of recovery, we initiated the experiments.

Drug infusions were performed using dental needles inserted 1.5 mm below the cannula end. A 0.5 μL/side volume was delivered over 1 min using 10 μL microsyringes (Hamilton, USA) connected to a polyethylene catheter in an infusion pump (Insight, Brazil). Drug flow was monitored via air bubble displacement in the catheter. Needles were removed 30 s post-infusion.

After experiments, rats were euthanized with ketamine (100 mg/kg) and xylazine (25 mg/kg). Methylene Blue (0.5 μL/side) was injected to verify the infusion site. Brains were sectioned at 100 μm using a vibratome (Leica, Germany), and animals were included in analyses only if Methylene Blue marked both DH sides.

**1.4 Viral infusion** **and chemogenetic inhibition**

AAV1-CD68-hM4Di-mCherry (Addgene, USA) was infused to induce hM4Di expression in microglia in the DH CA1. The virus at a titer of 3.3×10^12^ vg/mL was delivered in a 1.0 μL/side volume over 10 min, and needles were removed 5 min post-infusion. The experiments started 21 days post-surgery for viral expression, as described by Cardoso et al. [3].

At the end of the experiments, animals were deeply anesthetized and transcardially perfused with PBS and 4% paraformaldehyde (PFA). Brains were fixed in 4% PFA for 24 h, cryoprotected in 10%, 20%, and 30% sucrose solutions, frozen in dry ice, and sectioned at 40 μm on a cryostat (Leica, Germany). To confirm fluorescent labeling and cell specificity in the DH CA1 region, free-floating sections were incubated in blocking solution [0.3% Triton X-100, 1% BSA in TBS, 3 h at RT], followed by overnight incubation at 4 °C with a polyclonal goat anti-Iba-1 antibody (1:1000, FUJIFILM Wako, cat. no. 011-27991). The next day, sections were incubated for 2 h at RT with the corresponding chicken anti-goat secondary antibody (1:1000, Alexa Fluor™ 647, Thermo Fisher Scientific, cat. no. A-21469, USA). DAPI (final concentration 0.025 µg/mL; Sigma-Aldrich, cat. no. D9542) was added to the secondary antibody solution for nuclear staining. Sections were mounted on gelatin-coated slides using ProLong™ Diamond Antifade Mountant (Thermo Fisher Scientific, cat. no. P36970, USA).

Overview images of DH CA1 were acquired using a Leica TCS SPE confocal laser scanning microscope (Leica Microsystems, Germany) at 63× magnification (NA 1.3). The appropriate excitation and emission settings (wavelengths in nm) were configured as follows: 353/465 for DAPI, 587/610 for mCherry, and 650/673 for Iba-1 (Alexa Fluor™ 647). Stitched Z-stack images (20 steps) were obtained using the multidimensional acquisition function (1024 × 1024 pixels) and processed as maximum intensity projections using ImageJ (NIH, USA).

**1.5 Analysis of THC and its metabolites (11-OH-THC and THC-COOH)**

The blood was transferred into 2 mL polypropylene tubes containing spray-coated potassium-EDTA. Plasma was prepared by centrifugation at 1450 × g at 4 °C for 15 min. The DH was quickly removed on an ice-cold glass plate, according to [4]. Samples were frozen on dry ice and stored at −80 °C until analysis.

Plasma (750 μL) was transferred into 2 mL tubes, and proteins were precipitated with an ice-cold solution of acetonitrile (ACN) plus 1% Formic Acid (FA) in a plasma:ACN ratio of 1:3. The DHs were homogenized in the same solution. Plasma and DH were mixed for 2 min and centrifuged at 15000 × g at 4°C for 15 min. The supernatant was collected and transferred into a 2 mL tube. 1 mL of hexane was added, the solution was mixed, the organic phase was transferred into another tube, evaporated, and resuspended in 50 μL of methanol. The samples were homogenized in a vortex, incubated for 8 min at 25 °C in an ultrasound bath, then centrifuged for 3 min at 18900 × g, and transferred to HPLC vials for analysis. All samples were prepared according to [5]

The determination of THC and its metabolites, 11-hydroxy-THC (11-OH-THC) and 11-nor-9-carboxy-THC (THC-COOH) were investigated by high-performance liquid chromatography coupled to a mass spectrometer in tandem (HPLC-MS/MS). The HPLC apparatus (1200 Series, Agilent Technologies, Waldbronn-BW, Germany), coupled with a MS/MS system, was composed of a hybrid triple quadrupole/linear ion trap mass spectrometer (Q Trap 3200 Applied Biosystems/MDS Sciex, Concord-ON, Canada) operated in positive electrospray ionization mode (ESI). The separation was performed on a ZORBAX Eclipse Plus C18 column (50 x 2.1 mm, 1.8 mm; Agilent Technologies) at 30 ºC using a mobile phase composed of ACN/aqueous FA 0.1% (70/30, v/v) in isocratic elution. The flow rate was maintained at 350 μL min⁻¹ with a 5 μL injection volume. Analyst software version 1.5.1 was used to control the LC–ESI-MS/MS system and analyze data. The MS/MS parameters used were: capillary needle maintained at 4500 V; curtain gas at 10 psi; temperature at 450 ◦C; gas 1 and gas 2 at 50 psi; and CAD gas, low. Analytes (THC, 11-OH-THC, and THC-COOH) were monitored using Multiple Reaction Monitoring (MRM), and their MS parameters were set according to [6] (Table 1).

| Analyte | Q1 Mass (m/z) | Q3 Mass (m/z) | Dwell Time (ms) | DP (V) | EP (V) | CE (V) | | CXP (V) |
| --- | --- | --- | --- | --- | --- | --- | --- | --- |
| THC | 315.0 | 193.4 | 150 | 50 | 4.5 | | 30 | 3.0 |
|  | 315.0 | 123.2 | 50 | 55 | 3.7 | | 44 | 4.0 |
| 11-OH-THC | 331.0 | 193.3 | 500 | 45 | 4.0 | | 16 | 3.6 |
|  | 331.0 | 313.5 | 50 | 45 | 4.0 | | 16 | 3.6 |
| THC-COOH | 345.5 | 299.5 | 50 | 60 | 5.0 | | 30 | 5.1 |
|  | 345.5 | 327.5 | 150 | 60 | 5.0 | | 20 | 5.2 |

Table 1. Experimental details for LC-MS/MS acquisition [Pavlic et al. (6)].

Abbreviation: Q1 = quadrupole 1, Q3 = quadrupole 3, DP = declustering potential, EP = entrance potential, CE = collision energy, CXP = collision exit potential.

**Supplementary Figures**

**Figure S1.** Representative histological image showing the placement of infusion into the CA1 DH [adapted from (7)]

**Figure S2.** Effects of THC (0.002 mg/kg) treatment during fear memory reconsolidation in NOR learning in males and females. **A)** Experimental design. **B)** THC decreased the freezing time in Test A, confirming the impairment in fear memory reconsolidation in males [F_(1,16)_=11.01; *p*=0.004; η^2^=0.41]. **C)** No differences were observed between groups in the NOR test of males [t_16_=0.77; *p*=0.45; *d*=0.36]. n per group: VEH: 9, THC: 9. **D)** THC decreased the freezing time in Test A, confirming the impairment in fear memory reconsolidation in females during estrus and diestrus [F_(1,14)_=6.33, *p*=0.025; η^2^=0.31]. **E)** No differences were observed between groups in the NOR test of females [t_14_=0.91; *p*=0.38; *d*=0.45]. n per group: VEH: 8, THC: 8. Results are expressed as mean ± SEM and individual values. The * indicates *p* < 0.05 compared with the session control group.

**Figure S3**: Representative chromatograms obtained by HPLC-MS/MS in positive mode for transition m/z 331.0 à 313.5 (11-OH-THC). Samples: plasma and dorsal hippocampus (DH) from male (M) and female (F) rats, 30 and 60 minutes after i.p THC 0.002 mg/kg administration; blank (methanol). n=5 rats per group. THC metabolite 11-OH-THC was detected in all plasma and dorsal hippocampus (DH) samples, with a retention time of 3.6 minutes, which confirms the presence of THC. The THC per se was not detected (retention time of 3.2 minutes in the method).

**Figure S4:** Chromatographic peak area (m/z: 331.0 à 313.5) obtained for 11-OH-THC in plasma and in the dorsal hippocampus (DH) of male (blue) and female (pink) rats at 30 and 60 minutes after THC administration. Values are expressed as a percentage (left y-axis), with corresponding absolute responses shown on the right y-axis; the black line indicates the response for the blank sample (methanol) (0.28%).

**Figure S5.** Effects of THC (0.002 mg/kg) on microglial activity during fear memory reconsolidation in the PL cortex. **A)** Experimental design. **B)** Representative photomicrographs showing Iba-1+ cells at 20x magnification. Scale bar = 200 μm. **C)** In males, there is no difference in the Iba-1 fluorescence intensity in the PL cortex. [F_(2,16)_=0.60; *p*=0.56; η^2^=0.07]. n per group: Naive: 5, VEH: 7, THC: 7. **D)** In females, there is no difference in the Iba-1 fluorescence intensity in the PL cortex [F_(2,17)_=0.67; *p*=0.52; η^2^=0.07], suggesting an absence of microglial recruitment induced by fear memory retrieval or THC treatment. n per group: Naive: 6, VEH: 7, THC: 7. Results are expressed as mean ± SEM and individual values.

**References**

1. Meyer E, Rieder P, Gobbo D, Candido G, Scheller A, de Oliveira RMW, et al. Cannabidiol Exerts a Neuroprotective and Glia-Balancing Effect in the Subacute Phase of Stroke. Int J Mol Sci. 2022;23(21).

2.	Lisboa SF, Niraula A, Resstel LB, Guimaraes FS, Godbout JP, Sheridan JF. Repeated social defeat-induced neuroinflammation, anxiety-like behavior and resistance to fear extinction were attenuated by the cannabinoid receptor agonist WIN55,212-2. Neuropsychopharmacology. 2018;43(9):1924–33.

3.	Cardoso NC, Sohn JMB, Raymundi AM, Santos MR, Prickaerts J, Gazarini L, et al. Time-dependent fear memory generalization triggered by phosphodiesterase 5 inhibition during reconsolidation. Prog Neuropsychopharmacol Biol Psychiatry. 2025;137:111274.

4. Raymundi AM, Batista Sohn JM, Salemme BW, Cardoso NC, Silveira Guimarães F, Stern CA. Effects of delta-9 tetrahydrocannabinol on fear memory labilization and reconsolidation: A putative role of GluN2B-NMDA receptor within the dorsal hippocampus. Neuropharmacology. 2023;225.

5. Ruiz CM, Torrens A, Castillo E, Perrone CR, Cevallos J, Inshishian VC, et al. Pharmacokinetic, behavioral, and brain activity effects of Δ9-tetrahydrocannabinol in adolescent male and female rats. Neuropsychopharmacology. 2021 Apr;46(5):959-969.

6. Pavlic M, Innerhofer C, Pitterl F. Quantification of ∆9-tetrahydrocannabinol, 11-OH-THC, THC-COOH, hexahydrocannabinol, and cannabidiol in human plasma and blood by liquid chromatography-tandem mass spectrometry. J Anal Toxicol. 2025 Feb 15;49(2):85-95.

7. Paxinos G, Watson C. The rat brain in stereotaxic coordinates. 6. ed. Amsterdam Heidelberg: Academic Press; Elsevier; 2007.
